# Integration, not replacement: molecular methods and a new equilibrium in taxonomy

**DOI:** 10.1093/biosci/biag065

**Published:** 2026-05-21

**Authors:** Jan Kollár

**Affiliations:** Department of Ecology, Faculty of Science, Charles University, Viničná 7, CZ-128 00 Prague, Czech Republic

**Keywords:** biodiversity science, integrative taxonomy, molecular methods, natural history collections, research infrastructure

The rapid expansion of molecular methods since the 1990s has reshaped taxonomy. As sequencing became faster and cheaper, DNA-based approaches moved from niche applications to mainstream tools in systematics, phylogenetics, and biodiversity research. This transition is sometimes framed as a trajectory of replacement (Hebert et al. 2003), with morphology understood as a “legacy system” and organismal expertise as a craft at risk of extinction (Wheeler et al. 2004). That framing has influenced funding priorities and academic training, often favoring molecular infrastructure over long-term support for natural history collections and organismal education (Godfray 2002). However, large-scale bibliometric data suggest a different trajectory.

An analysis of more than 4 million Web of Science–indexed documents published between 1988 and 2023 across major eukaryotic lineages (and cyanobacteria) reveals a remarkably consistent pattern (figure. 1; Kollár et al. 2025). After steep growth following the adoption of molecular techniques, the proportion of studies incorporating molecular data does not continue to rise indefinitely. Across disparate taxonomic groups—including animals, fungi, land plants, and multiple protist lineages, as well as cyanobacteria—it stabilizes asymptotically, typically between ∼25 and 45% of published studies (with the notable exception of diatoms). Rather than continuing toward total dominance, molecular methods appear to converge on a stable equilibrium, often near one-third of the annual research output.

**Figure 1.**
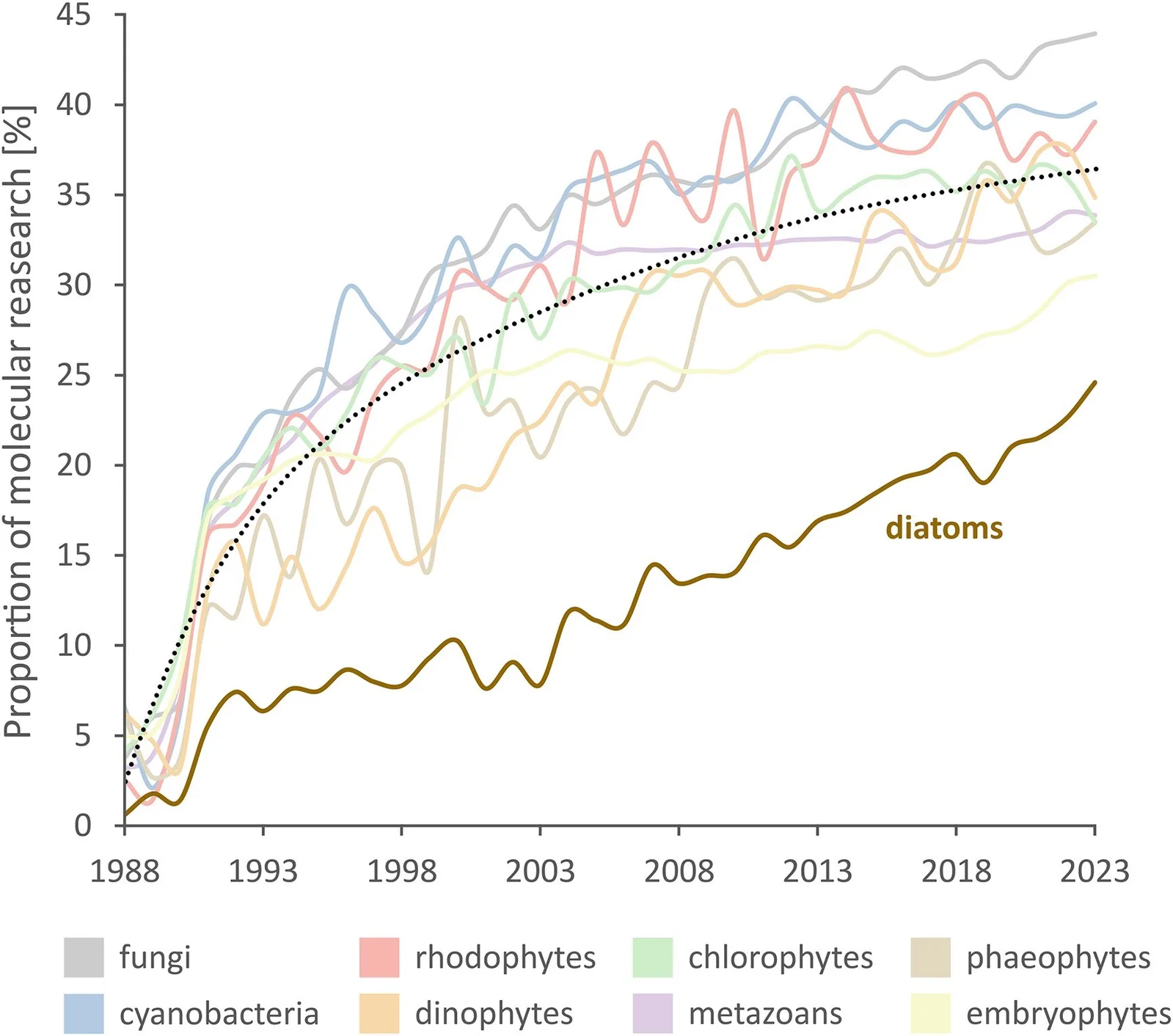
Long-term trends in the proportion of taxonomic research incorporating molecular data across major lineages (1988–2023). The annual share of molecular studies rose rapidly after the early 1990s, but in most major groups, it later slowed and approached an apparent asymptote. Individual lines represent different lineages, the diatom trajectory is shown separately, and the dotted line summarizes the average across the non-diatom groups depicted. Rather than continuing toward universal adoption, the use of molecular methods appears to stabilize across lineages, while diatoms remain below that broad range but continue to increase. Adapted from Kollár et al. (2025), with permission.

This recurring plateau is more consistent with complementarity than with succession. Rather than marking an incomplete transition toward molecular dominance, it suggests a durable division of labor in which molecular and organismal approaches remain mutually necessary (Will et al. 2005). This division of labor likely follows from the inherent strengths and limitations of DNA-based data. Molecular methods are exceptionally powerful for reconstructing phylogenetic relationships, detecting cryptic lineages, and scaling biodiversity analysis beyond the reach of traditional approaches. But biological diversity is expressed across multiple levels of organization, and sequence variation does not exhaustively capture phenotype, functional morphology, or ecological interactions among living organisms (Deans et al. 2015). For a substantial portion of taxonomic and ecological research, DNA-based data therefore function as a powerful diagnostic layer rather than a complete substitute for organismal observation. The observed equilibrium is thus not a temporary compromise, but a predictable outcome of what different kinds of evidence can and cannot reveal.

This interdependence is most evident in the continuing need for a physical and nomenclatural anchor. Molecular data do not exist in a biological vacuum. Their interpretability depends on robust reference frameworks of described species, type material, voucher specimens, and phenotypic context (Deans et al. 2015). Without that morphological and historical grounding, DNA sequences risk becoming “dark taxa”—informative, but semi-anonymous signals that remain only weakly connected to biological meaning (Page 2016). The molecular revolution has therefore not diminished the value of natural history collections. If anything, it has made them more important as reference systems and genomic archives. Weakening the infrastructure that supports organismal expertise therefore does not merely affect traditional taxonomy; it also reduces the interpretability and long-term value of molecular research.

If the observed equilibrium reflects the long-term structure of modern taxonomy, then the appropriate response is not to privilege one approach over another, but to sustain the conditions under which they can be combined. Training, collections, curation, and molecular capacity should not be treated as competing investments in a transitional phase, but as complementary components of a durable research infrastructure. The lesson is not that organismal evidence should resist molecular approaches, but that neither is sufficient on its own. The future of taxonomy lies in an integrative, polyphasic practice organized around the active combination of methods rather than expectations of replacement (Dayrat 2005).

Ultimately, the asymptotic stabilization of molecular methods should be read not as a failure of the molecular revolution, but as a sign of a maturing discipline. In an era of accelerating biodiversity loss, taxonomy can no longer be organized around a perceived opposition between molecular and organismal approaches, nor around the expectations of replacement. The task now is to train a new generation of researchers who are fluent across evidentiary traditions—equally capable of working with sequences, organisms, and collections. That, in turn, requires a shift in how the taxonomic enterprise is valued and supported, from a model of technological succession to one of durable integration (Pante et al. 2015). If organismal expertise is allowed to erode under the mistaken assumption that it is a legacy system, biodiversity science will lose the very context needed to interpret the molecular record. The challenge now is not to complete a replacement, but to build the infrastructure capable of sustaining integration.

## Conflict of interest

The author declares no conflicts of interest.

## Author Biography

Jan Kollár is a researcher at the University of Rostock, Germany, and was previously affiliated with Charles University, Prague, Czech Republic, where this manuscript was prepared. He studies species diversity in microbial eukaryotes, especially diatoms, with a focus on cryptic species diversity and integrative approaches that combine molecular and organismal data.
